# Mutations in THAP1/DYT6 reveal that diverse dystonia genes disrupt similar neuronal pathways and functions

**DOI:** 10.1371/journal.pgen.1007169

**Published:** 2018-01-24

**Authors:** Zuchra Zakirova, Tomas Fanutza, Justine Bonet, Ben Readhead, Weijia Zhang, Zhengzi Yi, Genevieve Beauvais, Thomas P. Zwaka, Laurie J. Ozelius, Robert D. Blitzer, Pedro Gonzalez-Alegre, Michelle E. Ehrlich

**Affiliations:** 1 Department of Neurology, Icahn School of Medicine at Mount Sinai, 1 Gustave L. Levy Pl, New York, NY, United States of America; 2 Departments of Pediatrics and Genetics and Genomic Sciences, Icahn School of Medicine at Mount Sinai, 1 Gustave L. Levy Pl, New York, NY, United States of America; 3 Bioinformatics Laboratory, Department of Medicine, Icahn School of Medicine at Mount Sinai, 1 Gustave L. Levy Pl, New York, NY, United States of America; 4 Raymond G. Perelman Center for Cellular & Molecular Therapeutics, The Children’s Hospital of Philadelphia, Philadelphia, PA, United States of America; 5 Black Family Stem Cell Institute, Icahn School of Medicine at Mount Sinai, New York, NY, United States of America; 6 Department of Neurology, Massachusetts General Hospital, 55 Fruit Street, Boston, MA, United States of America; 7 Department of Neurology, Harvard Medical School, Boston, MA, United States of America; 8 Departments of Pharmacological Sciences and Psychiatry, Icahn School of Medicine at Mount Sinai, 1 Gustave L. Levy Pl, New York, NY, United States of America; 9 Department of Neurology, Perelman School of Medicine at the University of Pennsylvania, Philadelphia, PA, United States of America; University of Minnesota, UNITED STATES

## Abstract

Dystonia is characterized by involuntary muscle contractions. Its many forms are genetically, phenotypically and etiologically diverse and it is unknown whether their pathogenesis converges on shared pathways. Mutations in *THAP1* [THAP (Thanatos-associated protein) domain containing, apoptosis associated protein 1], a ubiquitously expressed transcription factor with DNA binding and protein-interaction domains, cause dystonia, DYT6. There is a unique, neuronal 50-kDa Thap1-like immunoreactive species, and Thap1 levels are auto-regulated on the mRNA level. However, *THAP1* downstream targets in neurons, and the mechanism via which it causes dystonia are largely unknown. We used RNA-Seq to assay the *in vivo* effect of a heterozygote *Thap1* C54Y or ΔExon2 allele on the gene transcription signatures in neonatal mouse striatum and cerebellum. Enriched pathways and gene ontology terms include eIF2α Signaling, Mitochondrial Dysfunction, Neuron Projection Development, Axonal Guidance Signaling, and Synaptic LongTerm Depression, which are dysregulated in a genotype and tissue-dependent manner. Electrophysiological and neurite outgrowth assays were consistent with those enrichments, and the plasticity defects were partially corrected by salubrinal. Notably, several of these pathways were recently implicated in other forms of inherited dystonia, including DYT1. We conclude that dysfunction of these pathways may represent a point of convergence in the pathophysiology of several forms of inherited dystonia.

## Author summary

Dystonia is a brain disorder that causes disabling involuntary muscle contractions and abnormal postures. Mutations in *THAP1*, a zinc-finger transcription factor, cause DYT6, but its neuronal targets and functions are unknown. In this study, we sought to determine the effects of Thap1C54Y and ΔExon2 alleles on the gene transcription signatures at postnatal day 1 (P1) in the mouse striatum and cerebellum in order to correlate function with specific genes or pathways. Our unbiased transcriptomics approach showed that *Thap1* mutants revealed multiple signaling pathways involved in neuronal plasticity, axonal guidance, and oxidative stress response, which are also present in other forms of dystonia, particularly DYT1. We conclude that dysfunction of these pathways may represent a point of convergence on the pathogenesis of unrelated forms of inherited dystonia.

## Introduction

Dystonia is a brain disorder that causes disabling involuntary muscle contractions and abnormal postures. When this is the only feature, it is termed isolated dystonia. The pathogenic molecular mechanisms underlying the neuronal dysfunction that leads to dystonia remain to be elucidated and current treatments are unsatisfactory. The advent of next generation sequencing is rapidly expanding the list of genes causing isolated dystonia, including dominant mutations in *THAP1* (DYT6), *TOR1A* (DYT1), *GNAL* (DYT25), *ANO3* (DYT24), *CIZ1* (DYT23) and *TUBB4A* (DYT4), and recessive mutations in *HPCA* (DYT2), *COL6A3* (DYT27) and PRKRA (*DYT16*) [1–6], although some of these are still pending confirmation. Apart from rare inherited defects in dopamine synthesis [7], there is no known biological pathway that causally links genetic forms of dystonia. Phenotypic similarities between some inherited dystonias, including the most common DYT1 and DYT6, may suggest a shared underlying pathogenic mechanism. Uncovering such mechanisms would be a significant milestone, and potentially widely applicable for therapeutic development.

DYT6 is caused by mutations in *THAP1* [Thanatos-associated (THAP) domain-containing apoptosis-associated protein] [8,9], encoding a ubiquitously expressed transcription factor [10,11]. Similar to DYT1, caused by a mutation in *TOR1A* encoding TorsinA, the disease phenotype is restricted to the central nervous system despite widespread expression of the mutated protein. Disease-causing mutations in *THAP1* are dispersed throughout the coding regions, but most are missense and located in the DNA-binding domain (DBD) [12]. THAP domain DNA-binding activity is zinc-dependent, and the four metal-coordinating residues of the C2CH module are crucial for functional activity [13]. Nonsense mutations, equivalent to a null allele, likely result in the generation of small mRNA species that are subject to nonsense-mediated decay [8]. Little is known about the biological function of THAP1, particularly in neurons, although there is a neuron-specific DNA-binding THAP1-like-immunoreactive species [10]. There is also an alternatively spliced form lacking Exon2 which functionally does not substitute for the full-length protein [14] and is normally present at very low levels in the brain [15].

DYT6 is inherited in an autosomal dominant manner with reduced penetrance. Few brains from DYT6 patients have been examined and, to date, they do not exhibit any characteristic neuropathologic lesions [16]. Structural and functional neuroimaging in DYT6 manifesting and non-manifesting carriers (NMCs) demonstrates abnormalities in cerebello-thalamo-cortical and cortico-striato-pallido-thalamo-cortical pathways [17]. Genetically engineered mice with heterozygote *Thap1* mutations, either C54Y or ΔExon2, display structural abnormalities of the deep cerebellar nuclei and deficits on motor tasks without overt dystonia [18]. Both mutations are early embryonic lethal when homozygote [18], and in mouse embryonic stem cells, lead to decreased viability and neuroectodermal differentiation [14]. The cell cycle is the major dysregulated pathway that emerged from microarray assays of HUVECs with up- or down-regulation of THAP1 and of human lymphoblasts harboring a disease-associated intronic variant of *THAP1* [19,20], but was not enriched in ES cells [14].

To study the role of Thap1 in brain, we performed unbiased transcriptomic, RNA-Seq profiling of postnatal day 1 striatal and cerebellar tissue in two genetic mouse models of *THAP1*/DYT6 harboring mutations that alter the DNA binding domain, either (1) *Thap1*^*C54Y*^, a constitutive knock in (KI) of the C54Y causative mutation in the DNA binding domain (DBD) of *THAP1 and (2) Thap1*^*-*^, a constitutive knockout (KO) of exon 2 (ΔExon2) [18]. This was followed by functional studies to validate dysregulated molecular pathways with a focus on those that were either “top hits” and/or overlapped with other dystonias. We, and others, [21] have found that molecular abnormalities are present in the developmental stage, but their relationship to the emergence and persistence of the phenotype remains to be determined.

## Results

### Differential gene expression analysis in the striatum and cerebellum of *Thap1*^*+/-*^ and *Thap1*^*C54Y /+*^ vs wild-type mice

**Fig 1:**
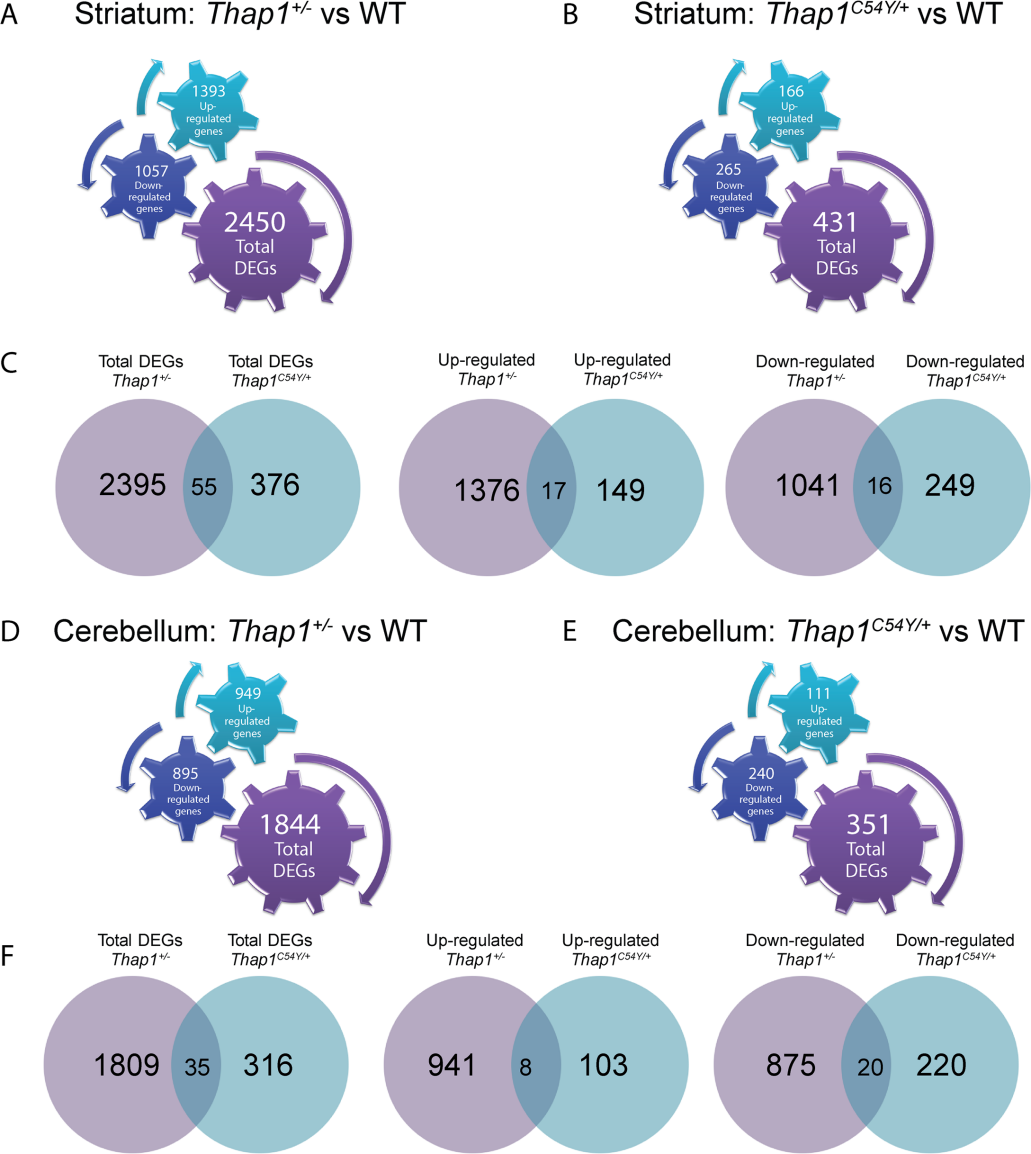
Global analysis of differential gene expression in striatum and cerebellum of *Thap1*^*+/-*^ or *Thap1*^*C54Y/+*^ vs WT. RNA-Seq was used to identify differentially expressed genes (DEGs) in the heterozygote *Thap1*^*+/-*^ and *Thap1*^*C54Y*^ P1 striatum and cerebellum as compared to WT. Diagrams show number of total DEGs as well as the number of up- or down-regulated genes in the **(A)** *Thap1*^*+/-*^ striatum vs WT **(B)** *Thap1*^*C54Y*^ striatum vs WT. **(C)** Venn diagrams show the number of overlapping DEGs (total, up-regulated or down-regulated) between *Thap1*^*+/-*^ and *Thap1*^*C54Y/+*^ relative to WT striatum. Diagrams show number of total DEGs as well as the number of up- or down-regulated genes in the **(D)** *Thap1*^*+/-*^ cerebellum vs WT **(E)** *Thap1*^*C54Y/+*^ cerebellum vs WT. **(F)** Venn diagrams show the number of overlapping DEGs (total, up-regulated or down-regulated) between *Thap1*^*+/-*^ and *Thap1*^*C54Y/+*^ relative to WT cerebellum. Cogged gears in panels A, B, D and E represent the number and the direction of the differentially expressed genes for each genotype and brain region, as follows: up-regulated genes (turquoise color, upward right arrow); down-regulated genes (dark blue color, downward left arrow); total [number] of genes (purple color, downward right arrow).

*Thap1*^*C54Y /+*^ KI mice carry a point mutation in one of three cysteine residues that are part of the zinc binding motif [19], altering binding of THAP1 to DNA [8,22]. *Thap1* KO mice, i.e. ΔExon2, deletes part of the DBD, and is referred to herein as *Thap1*^*+/-*^ [18]. We performed RNA-Seq analysis of cerebellar and striatal tissue dissected from postnatal day 1 (P1) *Thap1*^*+/-(ΔExon2)*^, *Thap1*^*C54Y/+*^ and wild-type (WT) controls (N = 4/genotype, all males). At this age, neurons outnumber glia and the limited studies in Ruiz *et al*. [18] showed greater changes in mRNA levels than in the adult. There was a higher number of differentially expressed genes (DEGs) in *Thap1*^*+/-*^ than in *Thap1*^*C54Y/+*^ in both structures (Fig 1 and S1–S4 Tables). 55 striatal DEGs overlapped between genotypes (Fig 1C, S5 Table) and 35 overlapped in the cerebellum (Fig 1F, S5 Table). The highest ranked DAVID Gene Ontology (GO) terms for the striatal overlapping DEGs were positive regulation of signal transduction, proteasome-mediated ubiquitin-dependent protein catabolic process and lipid storage, while those for overlapping DEGs in the cerebellum included cellular response to amino acid stimulus, and DNA-templated transcription. Among the overlapping DEGs, *Cdh4* and *Phf13* (up-regulated in striatum), *Wibg* and *Rsph1* (down-regulated in striatum), *Ppan* (differentially regulated in cerebellum), and *Nid2* (down-regulated in cerebellum) contain presumptive THABS motifs (S5 Table), supporting the notion that Thap1 may act as either an activator or repressor [13].

To determine if any of the DEGs are linked to dystonia or related disorders, we cross-matched them with the Emory University genetic dystonia panel (http://geneticslab.emory.edu/tests/MM550) and with the Mount Sinai Genetic Testing Laboratory Movement Disorders and Neuromuscular Disease Panel (S6 Table). We found 59 cross-matched striatal *Thap1*^*+/-*^ DEGs and 5 *Thap1*^*C54Y/+*^ DEGs. *Cryab* and *Fus* appeared in both sets. In cerebellum, we identified 54 cross-matched DEGs in the *Thap1*^*+/-*^ and 7 in *Thap1*^*C54Y/+*^, including 3 overlapping genes in both genotypes, *Thap1*, *Lama1* and *Sacs*.

As the RNA for this study was derived from whole tissue, we utilized an RNA-sequencing transcriptome and splicing database annotating glia, neurons, and vascular cells from the mouse brain [23], in order to investigate the cell subtype origin of the DEGs in the striatum and cerebellum of the *Thap1*^*+/-*^ or *Thap1*^*C54Y/+*^ vs WT mice (S7 Table). The largest cell-type fraction of both the up- and down- regulated genes in the *Thap1*^*+/-*^ striatum were neuronal, while the greatest fraction of the up- and down- regulated genes in the cerebellum were expressed in endothelial and oligodendrocyte progenitor cells, respectively. The largest cell-type fraction of both the up- and down- regulated genes in the *Thap1*^*C54Y/+*^ striatum were neuronal and astrocytic in origin, while most of the up- and down- regulated genes in the cerebellum were derived from endothelial and microglial cells (S7 Table). It should be noted that P1 mice will have a greater enrichment for cell growth and gene regulatory pathways. Zhang *et al*. [23] showed that oligodendrocyte-lineage cell isolation does not occur until P17, the earliest time point when the full collection of oligodendrocyte-lineage cells is present. Therefore, one potential limitation of this study is age at which the transcriptomes were assayed but it is also a strength in terms of looking at altered pathways during this critical period.

**Fig 2:**
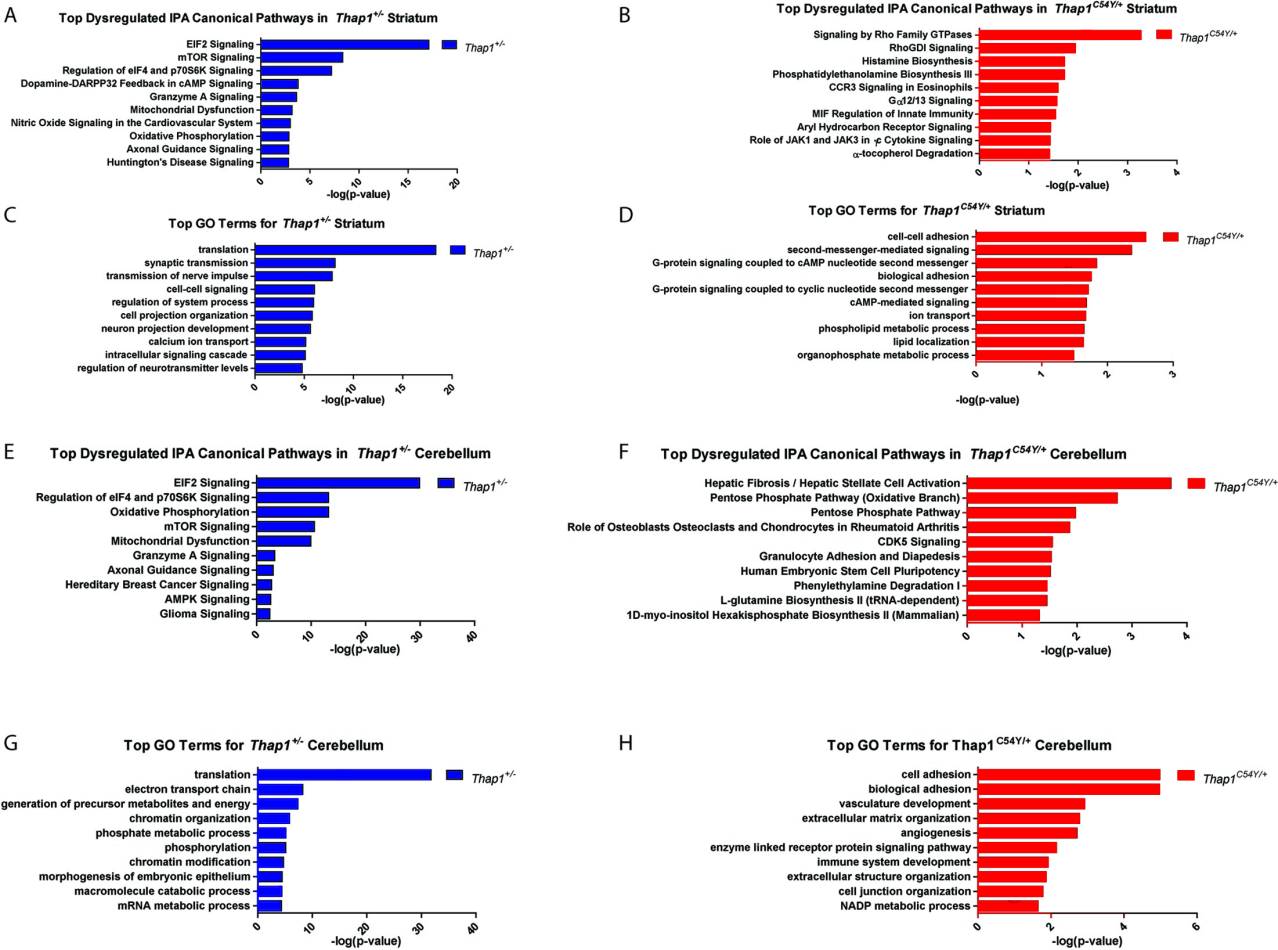
Top canonical pathways and gene ontology terms enriched in striatum and cerebellum of *Thap1*^*+/-*^ and *Thap1*^*C54Y/+*^ relative to WT. **(A,B)** Top canonical pathways as determined by IPA analysis, and **(C,D)** DAVID Gene Ontology (GO) terms show an enrichment of biological process based on the list of significant DEGs (DEseq p < 0.05) in the striatum of *Thap1*^*+/-*^ and *Thap1*^*C54Y/+*^ relative to WT. **(E,F)** Top canonical pathways and **(G,H)** DAVID GO terms based on the list of significant DEGs in the cerebellum of *Thap1*^*+/-*^ and *Thap1*^*C54Y/+*^ relative to WT.

Ingenuity Pathway Analysis (IPA) and DAVID GO terms identified biological functions and pathways enriched in DEGs from each dataset. The highest ranked IPA canonical pathways and GO Terms for each set of DEGs are shown in Fig 2 and S1 and S2 Tables. There were marked genotype-dependent differences, but there were overlaps between striatum and cerebellum within each genotype. Based on what were either the most significantly enriched pathways and terms, and/or those which are related to identified abnormalities in DYT6 and DYT1 models, we chose to functionally explore the eIF2α pathway, neuron projection development, synaptic plasticity [long term depression (LTD) and potentiation (LTP)], and mitochondrial Complex I.

To determine if any of the DEGs from the RNA-Seq datasets were bound directly by THAP1, we compared them against two publicly available THAP1 ChIP-Seq datasets, one from human ENCODE K562 cells, and the other from mouse ES cells [14]. The *Thap1*^*+/-*^ cerebellum had the greatest number of overlapping genes when compared to both ChIP-Seq datasets, with a total of 32 overlapping genes when compared to the mouse ES cell data and 39 overlapping genes when compared to the K562 dataset (S11 Table). The highest ranking biological functions enriched in the 32-member gene set as identified by DAVID GO are: cellular macromolecules metabolic process, ribosomal small subunit assembly, and negative regulation of protein kinase activity (S11 Table). The highest ranking biological functions in the 39-member gene set as identified by DAVID GO are: cellular process, cellular metabolic process and gene expression (S11 Table).

### *Thap1* mutant mice show evidence of abnormal eIF2α signaling

The eIF2α signaling pathway was one of the top differentially regulated signaling pathways in the striatum and cerebellum of *Thap1*^*+/-*^ mice. The eIF2α pathway is a key effector of the cellular response to several stressors, including the accumulation of misfolded proteins in the endoplasmic reticulum (ER), and was linked to TorsinA function soon after *TOR1A* was identified as the causative gene in DYT1 [24–26]. DYT16 is caused by mutations in *PRKRA*, a stress-activated modulator of the eIF2α kinase PKR, with evidence of abnormal phosphorylation of PKR and eIF2α in patient fibroblasts under ER stress [27,28]. In addition, a proteomics-based study identified abnormal eIF2α pathway activation in DYT1 mouse and rat brains, which correlated with assays in human brains [24]. Therefore, given the previous evidence suggesting a role of eIF2a signaling dysregulation in dystonia, and our own RNA-seq data, we investigated UPR genes and proteins to assay the baseline status of the UPR in Thap1 recombinant mice.

**Fig 3:**
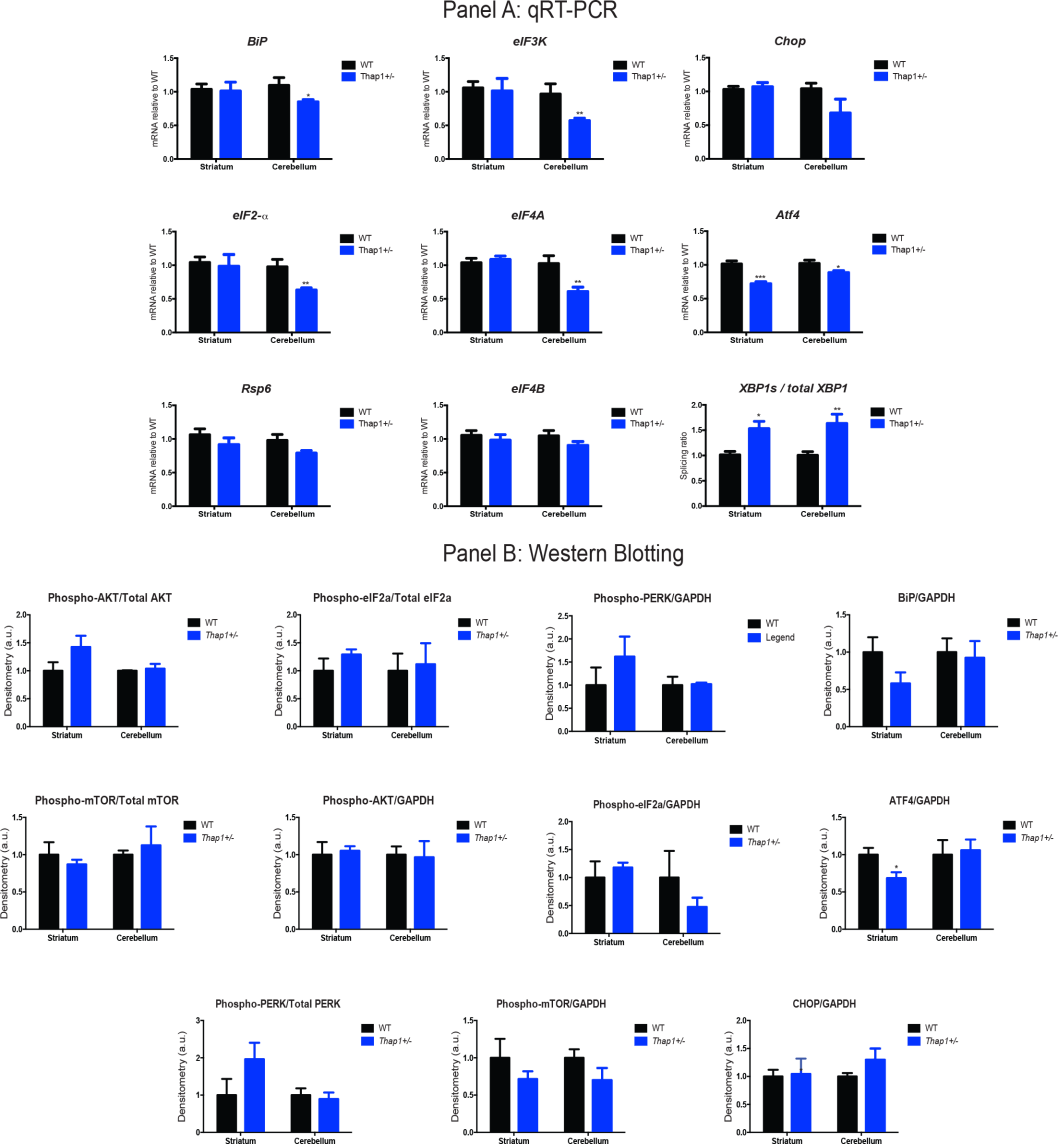
Key constituents of the eIF2α signaling pathway are down-regulated in the brains of *Thap1*^*+/-*^ mice as compared to WT. **(A)** mRNA expression gene profiles of key genes from the eIF2α signaling pathway were assayed with quantitative real-time PCR (RT-qPCR) using cerebellar samples. Data normalized relative to WT. Data are presented as means ± Standard Error of the Mean (SEM); n = 8 for each genotype and brain region with separate littermate WT controls, *Student’s t test* (*p<0.05; ** p<0.01; *** p<0.005). See also S9 Table. **(B)** Levels of protein expression in the striatum and cerebellum of P1 *THAP1*^*+/-*^ mice relative to WT were assayed by western blot. Densitometry measurements (arbitrary units) are normalized to the housekeeping gene GAPDH, or for phosphoproteins, relative to GAPDH and their respective holoprotein level. Data are presented as means ± SEM; n = 4 for each genotype and region with separate, littermate WT controls, *Student’s t test*. *p<0.05. See also S9 Table.

Initially, we assayed the relative mRNA levels for members of the eIF2α signaling pathway in P1 *Thap1*^*+/-*^ cerebellum and striatum by RT-qPCR. We assessed changes in genes known to play a role in UPR or eIF2α signaling pathways using DEGs found to be dysregulated directly from the RNA-seq analysis (*eIF3K*, *eIF2a*, *eIF4A*, *eIF4B*), as well as upstream and downstream mediators of eIF2α (*BiP*, *Chop*, *Rsp6*, *XBP1s*, *and total XBP1*) in Fig 3A. There was dysregulated expression of most components of this signaling pathway in cerebellum (S9 Table), and in the *Thap1*^*+/-*^ striatum significant differences were observed in *Atf4* expression, and *XBP1s/total XBP1* ratios (Fig 3, S9 Table). These data show that there are baseline abnormalities of the eIF2α signaling pathway; however, their contribution to the genotype-dependent phenotypes remains to be determined.

The eIF2α signaling pathway is involved in translational regulation, and notably, the DEGs were enriched for a second translational control pathway mediated by mTOR. These kinase cascades regulate protein function via phosphorylation and protein levels. We assayed components of both pathways at basal level by western blotting of protein lysates derived from P1 *Thap1*^*+/-*^ striatum and cerebellum. Consistent with the RNA-Seq and RT-qPCR data, the key effector of the eIF2α pathway, ATF4, was reduced by 25% in the striatum (Fig 3, right panel). Overall, the data lend further support to the presence of dysfunction of the eIF2α pathway in DYT6 brain. Notably, screening designed to identify genes implicated in the response to ER stress in human B cells via genetic interactions identified THAP1 as the top hit and all changes were at the level of protein interactions [29].

**Fig 4:**
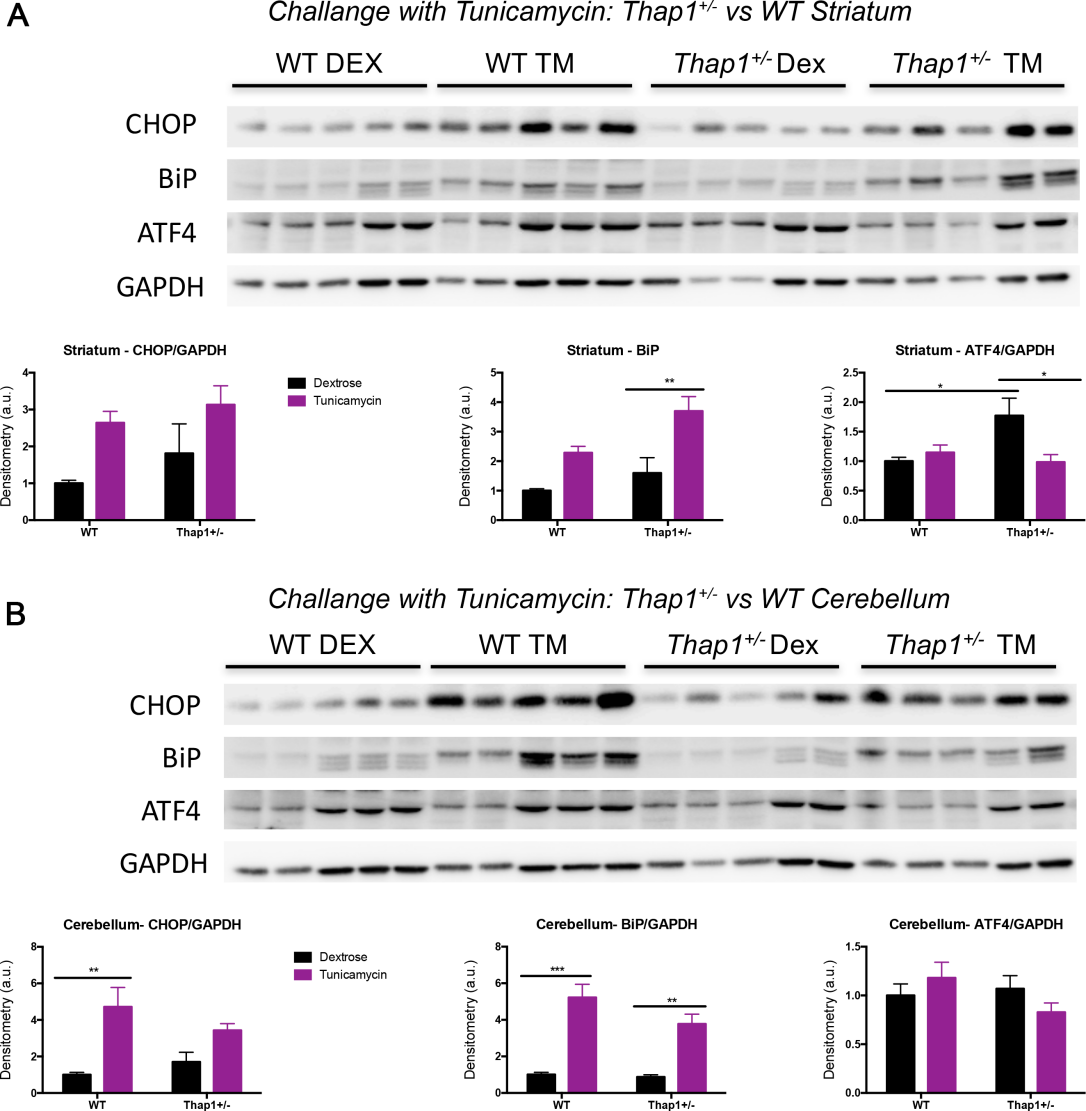
Tunicamycin challenge in P4 *Thap1*^*+/-*^ and WT pups increases eiF2α signaling pathway proteins and reveals genotype-dependent differences in striatum and cerebellum. Western blot analysis of **(A)** striatal and **(B)** cerebellar lysates from *Thap1*^*+/-*^ and WT littermates for BiP, ATF4 and CHOP were performed 24 hrs after subcutaneous tunicamycin (TM) diluted in 150mM dextrose (or dextrose-only control; DEX). Protein expression levels represent normalization to the housekeeping gene GAPDH. Data are presented as means ± SEM; n = 5 for each genotype and region, data normalized to WT (dextrose-only) controls. Statistical differences were assessed by two-way ANOVAs with Tukey’s *post hoc* tests. *p < 0.05; **p < 0.01; ***p < 0.001. See also S9 Table.

**Fig 5:**
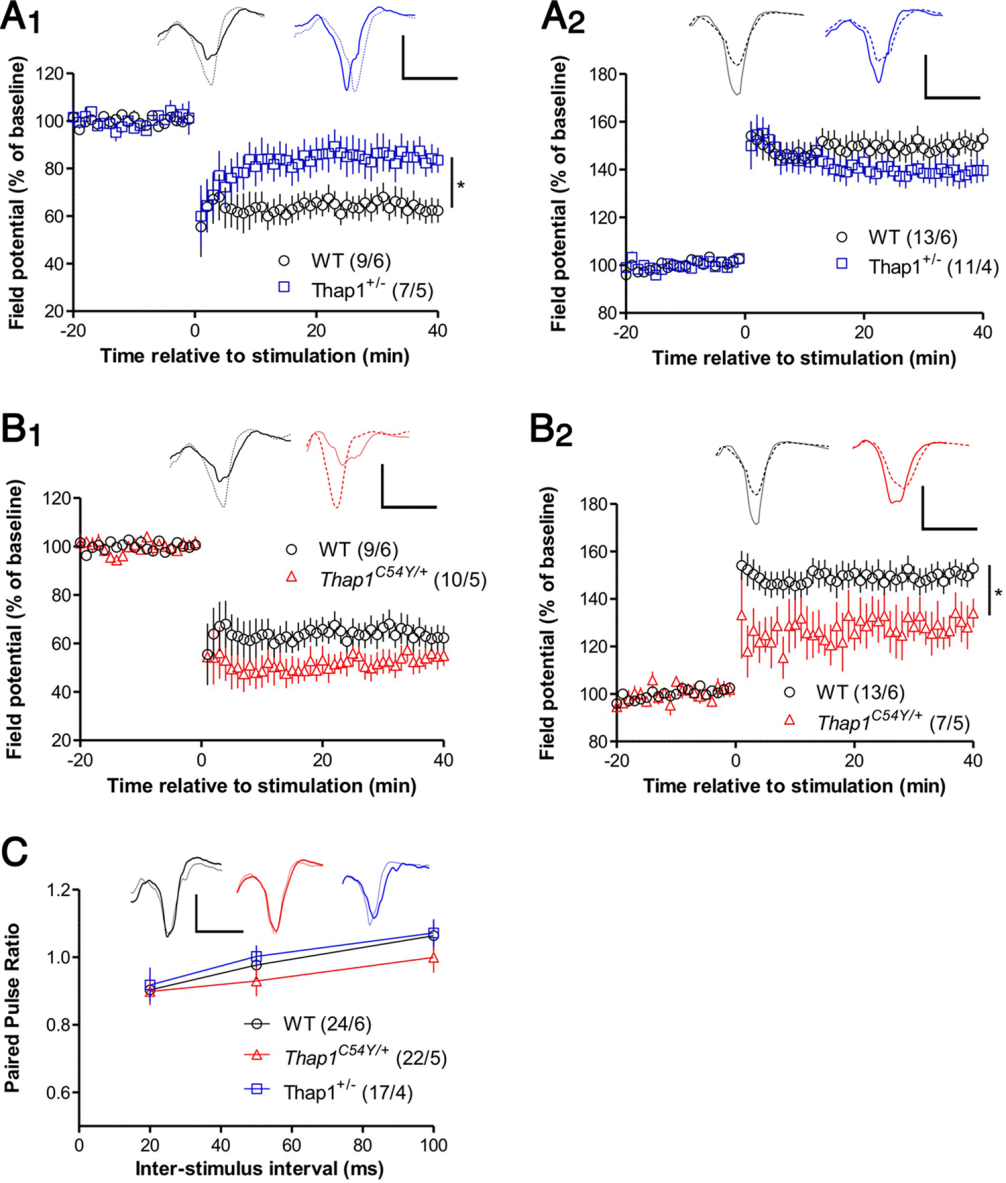
Cortico-striatal synaptic plasticity is altered in *Thap1*^*+/-*^ and *Thap1*^*C54Y/+*^ derived slices. **(A)** *Thap1*^*+/-*^ mice are deficient in synaptically-induced LTD in dorsolateral striatum compared to wildtype controls (A_1_; *p* < .05), while LTP in the dorsomedial region is intact (A_2_). Representative excitatory postsynaptic potential (EPSP) traces in this and panel B were averaged over the baseline period (thin line) and over the final 5 min of recording (thick line), color coded to the graph. Calibration for these and all other traces: 1 mV / 5 ms. **(B)** In *Thap1*^*C54Y/+*^ mice, LTD was not significantly reduced (B_1_), but LTP was deficient (B_2_; p < .05). Note that wildtype data are the same as for panel A, and that all genotypes were analyzed together. **(C)** Paired-pulse ratio was not altered in *Thap1*^*+/-*^ and *Thap1*^*C54Y/+*^ mice. The traces show averaged EPSPs recorded at inter-stimulus interval = 50 ms (thin and thick lines show responses to first and second stimuli, respectively). All graphs show group means ± SEM, and the number of slices/mice for each group are shown in parentheses. Data were analyzed by ANOVAs performed over the final 5 minutes of recording (panels A and B) or on averaged paired-pulse data for each interval (panel C), followed where appropriate by Newman-Keuls *post-hoc* tests. *p<0.05 See also S9 Table.

**Fig 6:**
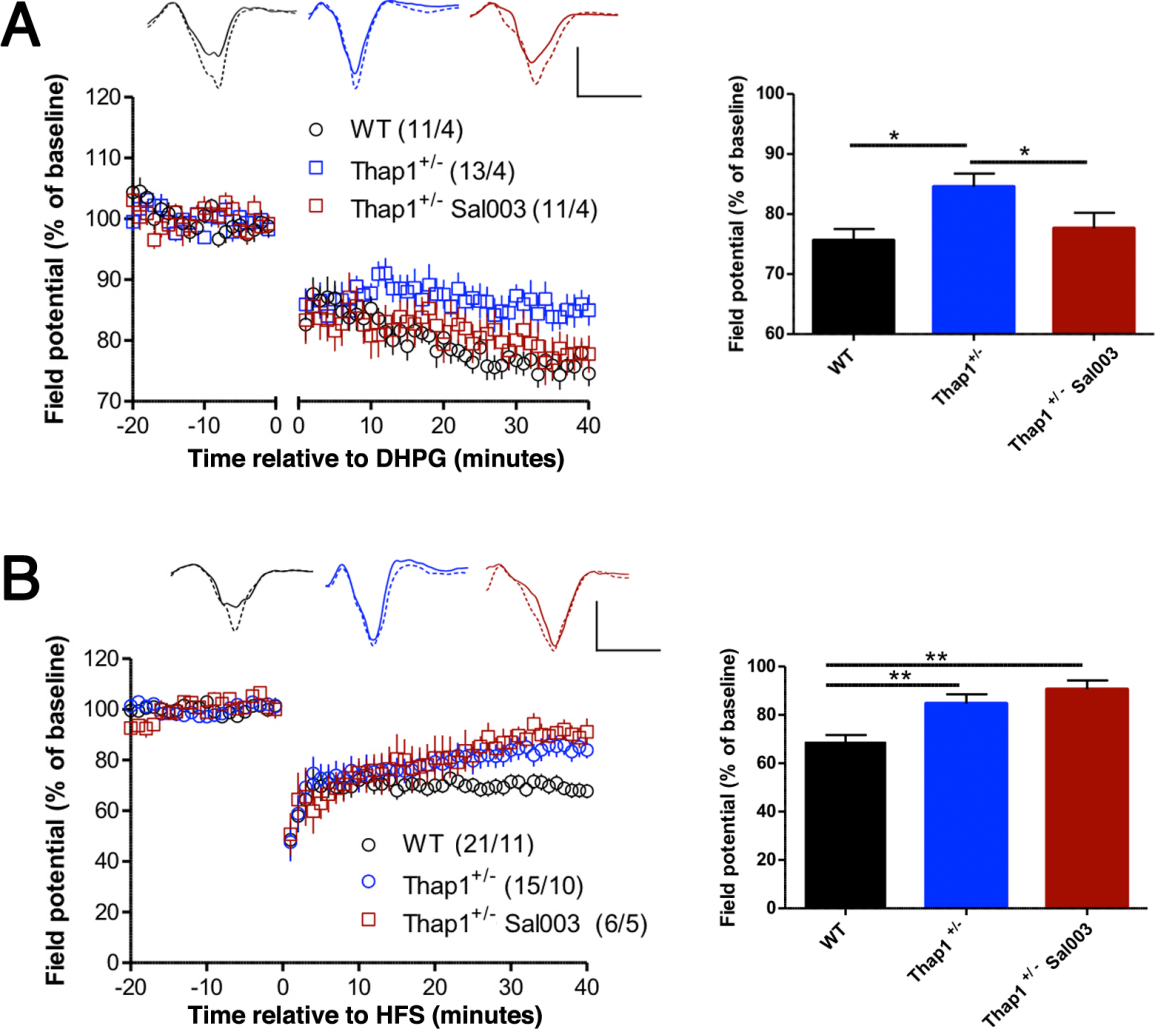
Inhibition of eIF2α phosphatase rescues mGluR-LTD, but not synaptically-induced LTD. The summary graphs in the right panels show mean ± SEM for the final 5 min of recording. **(A)** LTD after treatment with group 1 agonist DHPG (100 μM, applied during the gap in the graph) was reduced in *Thap1*^*+/-*^ slices, and pretreatment with Sal003 (20 μM) (eIF2α phosphatase inhibitor) rescued LTD in *Thap1*^*+/-*^ slices. **(B)** In high frequency stimulation (HFS)-induced LTD, Sal003 (10 μM) failed to reverse the deficit observed in *Thap1*^*+/-*^ slices. Numbers in parentheses indicate number of slices/number of mice. Representative traces are shown during baseline period (solid lines) and at the end of the recording period (dashed lines). Calibrations: 1 mV / 5 ms. Asterisks indicate *p* < .05 (*) or p < .01 (**) by ANOVAs followed by Newman-Keuls *post-hoc* tests. See also S9 Table.

**Fig 7:**
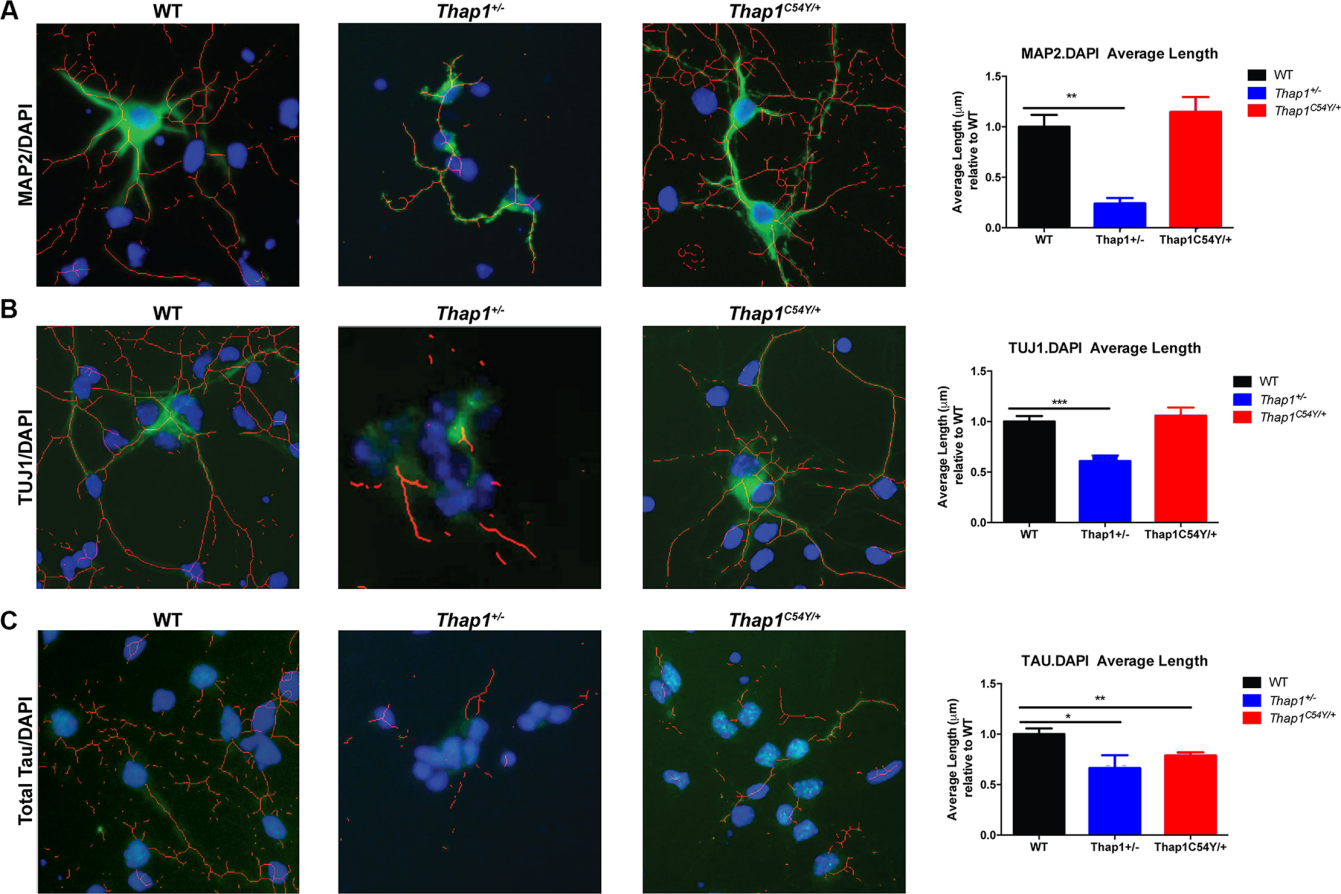
Neurite length in *Thap1*^*+/-*^ and *Thap1*^*C54Y/+*^ E16 primary striatal neurons *in vitro* is decreased relative to neurons from WT mice. Traces and measures of neurite length using NeuriteTracer show that *Thap1*^*+/-*^ striatal neurons have shorter processes as compared to WT as calculated after immunostaining with **(A)** MAP2, **(B)** TUJ1 or **(C)** Total Tau. *Thap1*^*C54Y/+*^ striatal neurons exhibit a milder phenotype and only have shorter axonal processes (Tau immunostaining) as compared to WT. The total number of nuclei (DAPI stain) was independent of genotype after plating equal numbers of neurons in all wells. Neurons were derived from 3 independent platings. Data are presented as means ± SEM, minimum of 25 neurons per well. Statistical differences were assessed by ANOVA with Student’s *post hoc* t-test. *p < 0.05; **p < 0.01; ***p < 0.001. See also S9 Table.

To assay the function of the eIF2α pathway in the presence of a *Thap1* mutation, we challenged P4 *Thap1*^*+/-*^ and WT pups with tunicamycin, which induces ER stress and the unfolded protein response (UPR) in liver, cerebral cortex, and cerebellum at this age [30]. There was a clear engagement of the main ER chaperone, BiP, in striatum of *Thap1*^*+/-*^ mice, and cerebellum of both genotypes (Fig 4). We detected genotype-dependent differences in the expression levels of ATF4 at basal level (dextrose-only controls) in the striatum of *Thap1^+/-^* mice as compared WT mice, and the most notable genotype-dependent difference following challenge with tunicamycin was a decrease in ATF4 striatal expression in *Thap1*^*+/-*^ but not in WT mice (Fig 4). No differences were observed amongst the different groups when we assessed p-eIF2α/eIF2α and p-eIF2α/GAPDH expression levels (S3 Fig). Therefore, we could not find consistent up or down changes in the UPR with Thap1 baseline status and tunicamycin treatments. Nonetheless, our data suggest a dysregulation of the eIF2α signaling pathway. Therefore, we went on to perform physiologic/functional experiments in Figs 5–7.

IPA pathways related to oxidative stress (i.e. Mitochondrial Dysfunction and Oxidative Phosphorylation) were significantly dysregulated in *Thap1*^*+/-*^ striatum and cerebellum, and abnormalities in these pathways can contribute to the UPR and to synaptic plasticity [31,32]. Specifically, mitochondrial complex I deficiency (OMIM:252010_3) was enriched (FDR < 0.05) in *Thap1*^*+/-*^ cerebellum using Harmonizome [33]. However, we found no genotype-dependent differences in complex I activity in either the striatum (t = 0.89, df = 5, p = 0.42) or cerebellum (t = 0.36, df = 5, p = 0.74).

### Synaptic plasticity is impaired in *Thap1*^*+/-*^ and *Thap1*^*C54Y/+*^ mouse striatum

Synaptic plasticity is a specific neuronal function predicted by IPA and GO to be disrupted in the striatum of Thap1+/- mice, particularly long-term depression (LTD) and the related phenomenon of synaptic depotentiation (S1 Table). This association is again reminiscent of the deficit in striatal LTD in mouse models of DYT1 [34,35], suggesting that this might be a convergent feature among different types of dystonia and might even be related to dysfunction of the eIF2α pathway [26]. Although differential gene expression for *Thap1*^*C54Y/+*^ striatum was less predictive of a synaptic plasticity phenotype (S2 Table), we tested whether persistent synaptically-induced forms of plasticity at glutamatergic synapses–both LTD and long-term potentiation (LTP)–were altered in the striatum of both mouse lines. Extracellular recordings in acute slices from *Thap1*^*+/-*^ mice stimulated with high-frequency synaptic stimulation (HFS) revealed greatly reduced LTD, similar to that reported for DYT1 mouse models (Fig 5A_1_, S9 Table). In contrast, LTP was normal in *Thap1*^*+/-*^ striatum (Fig 5A_2_). The plasticity phenotype was different for the *Thap1*^*C54Y/+*^ mice: LTD showed a non-significant trend towards enhancement (Fig 5B_1_, S9 Table), but LTP was reduced (Fig 5B_2_, S9 Table).

The potential for plasticity at inhibitory synapses could distort the interpretation of our striatal field recordings. For example, reduced LTD at excitatory synapses is also consistent with increased GABA-A mediated currents. To address this possibility, we repeated the LTD experiment in the presence of the GABA-A antagonist gabazine (S2 Fig), and confirmed that LTD was reduced in slices from *Thap1*^*+/-*^ mice. Moreover, the *Thap1*^*C54Y/+*^ mutation led to significantly enhanced LTD, in agreement with the trend observed in the absence of gabazine (Fig 5B_1_, S9 Table).

These results indicate that synaptic plasticity is susceptible to the deletion of *Thap1* Exon2 or expression of a DYT6-related mutation of *Thap1*, while the specific nature of the disruption differs between these two manipulations. Note that paired-pulse ratio was not affected in either the *Thap1*^*+/-*^ or *Thap1*^*C54Y/+*^ striatum (Fig 5C, S9 Table), indicating that presynaptic function was normal. However, basal synaptic efficiency was enhanced in *Thap1*^*C54Y/+*^ mice [stimulus strength to evoke 1 mV field excitatory postsynaptic potential (EPSP): 0.88 ± 0.11 μA (n = 24) for wildtype, 0.87 ± 0.10 μA (n = 21) for *Thap1*^*+/-*^, and 0.49 ± 0.05 μA (n = 23) for *Thap1*^*C54Y/+*^; S9 Table]. The increased efficiency in *Thap1*^*C54Y/+*^ striatum would be consistent with elevated synapse number. Alternatively, postsynaptic function could be up-regulated in *Thap1*^*C54Y/+*^ mice. Since striatal LTP at excitatory synapses is expressed postsynaptically [36], basal upregulation of postsynaptic function might limit the extent to which these synapses can be further potentiated, consistent with the reduced LTP seen in these mice.

These extracellular recordings reveal plasticity phenotypes that must be expressed by a substantial fraction of MSNs. Future patch-clamp experiments, performed on MSNs expressing either the D1 or D2 receptor, will be useful to determine if the *Thap1^+/-^* and *Thap1^C54Y/+^* mutations differentially affect LTD or LTP in MSN subtypes.

### *Thap1*^*+/-*^ deficiency in mGluR-LTD is normalized by inhibiting the eIF2α phosphatase

Normal regulation of eIF2α by phosphorylation is required for multiple forms of LTD. In the striatum, inhibition of the eIF2α kinase PERK prevents synaptically-induced LTD [26], and eIF2α has been implicated in hippocampal LTD induced by pharmacological stimulation of metabotropic glutamate receptors (mGluR-LTD) [37,38]. To test whether the LTD deficits in *Thap1*^*+/-*^ striatum could be due to dysregulation of eIF2α, we evaluated if abnormal LTD were rescued by Sal003, a selective inhibitor of the eIF2α phosphatase (Fig 6). We found differential effects of Sal003 on mGluR-LTD (induced by the group 1 agonist DHPG) and synaptically-induced LTD (induced by high-frequency stimulation; HFS). Sal003 restored mGluR-LTD to wildtype levels (Fig 6A, S9 Table), in agreement with the finding that eIF2α phosphorylation is required for this form of plasticity in the hippocampus [37]. However, Sal003 had no effect on HFS-induced LTD (Fig 6B, S9 Table). This finding contrasts with the ability of Sal003 to restore LTD in *DYT1* mutant mice [26], indicating that *Thap1* deletion can interfere with LTD by affecting signaling mechanisms in addition to eIF2α dysregulation. Since HFS-induced LTD in striatum depends on the synaptic activation of mGluRs [39,40], it is possible that, independent of its effect on eIF2α signaling,*Thap1* deletion interferes with the ability of HFS to activate postsynaptic mGluRs. In fact, we identified numerous dysregulated genes that participate in synaptic transmission and conduction of nerve impulses in *Thap1*^*+/*-^ striatum (S1 Table).

### Neurite outgrowth is decreased in *Thap1*^*+/-*^ and *Thap1*^*C54Y/+*^ striatal neurons *in vitro*

Neurite development was another enriched pathway identified across regions in *Thap1*^*+/-*^ mice. TorsinA has a proposed role in neuritogenesis, and diffusion tensor imaging abnormalities detected by MRI are seen in different brain regions of patients with DYT6, DYT1 and other dystonias [41–43]. Signaling by the Rho family of GTPases is the top pathway in the *Thap1*^*C54Y/+*^ striatum, and this pathway plays a critical role in neuritogenesis and axonal pathfinding [44]. To investigate the influence of Thap1 on this process, we assayed neurite development in striatal MSNs *in vitro*. We cultured striatal E16 (embryonic day 16) neurons from individual *Thap1*^*+/-*^ and WT embryos and quantified neurite length after 24 hours. *Thap1*^*+/-*^ striatal neurons exhibited shorter processes as compared to WT controls. The phenotype was present but less severe in *Thap1*^*C54Y/+*^ striatal neurons (Fig 7, S9 Table). The total number of cells was equal between the genotypes, suggesting normal survival of the heterozygote mutant neurons following plating at equal densities.

## Discussion

We used an unbiased RNA-Seq approach to identify dysregulated genes and pathways in mice harboring either a *Thap1* C54Y (disease causing) or a ΔExon2, i.e. null allele. A major goal was to determine if these pathways and resultant phenotypes overlap with abnormalities observed in other dystonias, particularly DYT1 [45], due to their clinical, electrophysiological, structural and functional neuroimaging similarities. RNA-Seq was performed in two brain regions, striatum and cerebellum, consistently implicated in dystonia pathogenesis [46–50], and at a developmental time point when Thap1 levels and transcriptional abnormalities peak [10,15,18], supporting the notion that dystonia is a developmental disorder [51,52]. These initial experiments yielded data consistent with the proposed hypothesis, further supported by ensuing functional validation.

### *Thap1*^*+/-*^ and *Thap1*^*C54Y/+*^ mice are not equivalent

The data herein support our previous conclusion that the C54Y mutation does not equal a DNA-binding loss-of-function mutation. First, there were a far greater number of DEGs in *Thap1*^*+/-*^ than *Thap1*^*C54Y/+*^ mice (Fig 1). Second, fold changes were overall lower in *Thap1*^*C54Y/+*^ in comparison to *Thap1*^*+/-*^ (S5 Table). Third, only a small number of DEGs were altered in both genotypes (S5 Table). For both mutations, the log2 values are low for what would be predicted for a transcription factor [53], perhaps in the presence of the C54Y mutation due to compensatory auto-up-regulation of *Thap1* [18,54]. Reports of dystonia patients with homozygous *THAP1* mutations with non-manifesting heterozygous parents [55,56] highlight dosage dependent effects of *THAP1* mutations.

*THAP1* mutations occur in all domains of the protein, but genotype-phenotype correlations have proven difficult to establish due to the small number of patients with each mutation. The C54Y protein, unlike the WT protein, does not bind to the *Tor1a* promoter [22], but it may bind DNA at other sites and perhaps aberrantly expands DNA binding beyond THAB motifs [57] and/or alter cofactor binding [58]. Thus, some DYT6 mutations represent a partial or total loss of function, whereas others could lead to a combination of haploinsufficiency and gain of function, accounting for the genotype-dependent DEG disparity. Shared transcriptomic and phenotypic features, despite the many differences between mice carrying the C54Y and null alleles, also support this possibility. Thus, there was overlap of key disordered biological processes and biofunctions in the striatum and cerebellum between genotypes. These results indicate that the choice of animal model must be carefully considered in DYT6 research, as different mutations can yield divergent results, although sometimes leading to dysregulation of the same pathways and processes.

### EIF2α signaling dysfunction: A link between DYT6 and other dystonias?

These new data together with published DYT1 studies [24,26], suggest a point of convergence of neuronal dysfunction on the eIF2α pathway in DYT6 and DYT1. To a lesser extent, other translational control pathways (mTOR and eIF4/p70S6K) are also implicated in the dysregulation produced by mutations in *Thap1*. Notably, as a transcription factor, THAP1 regulates *TOR1A* transcription in artificial systems, but this has not been verified *in vivo* [18,22,59].

A proteomics-based study identified abnormal eIF2α pathway activation in DYT1 mouse and rat brain, which correlated with assays in human brain [24]. A second group designed an RNAi-based functional genomic screening in HEK293T cells that also implicated the eIF2α pathway in DYT1 biology. Moreover, pharmacological manipulation of eIF2α signaling restored absent cortico-striatal LTD in DYT1 knock-in mice [26]. Together, these reports support the presence of abnormal eIF2α signaling in DYT1 brain and its possible causal link to DYT1 synaptic defects. EIF2α signaling provides a potential point of merger with another, rarer form of primary dystonia. DYT16 is caused by mutations in *PRKRA*, an activator of the eIF2α kinase PKR, with evidence of abnormal eIF2α phosphorylation in patient fibroblasts [27,28]. Coding variants in ATF4, a direct target of eIF2α, were found in patients with focal dystonia [26] and lastly, a recent gene-expression analysis in adult cerebellar tissue from a mouse model of DYT11 dystonia also identified genes associated with protein translation among the top down-regulated mRNAs [60].

We report eIF2α-pathway-related molecular and electrophysiological findings in DYT6 mice that have some similarities with those in DYT1, including abnormalities in baseline ATF4 and in LTD. Taken together, these reports suggest that efforts in dystonia research should include the unravelling of the mechanisms underlying these observations, addressing causality and reversibility.

### *Thap1* mutations alter synaptic plasticity and neuritogenesis

Multiple dysregulated pathways highlighted in the GO and IPA analyses of the RNA-Seq data may contribute to the deficits in synaptic plasticity and neuritogenesis described herein. These include eIF2α signaling, which in addition to being a key component of ER stress responses, regulates important physiological events under homeostatic conditions, including synaptic plasticity [61–65]. ATF4 plays a specific role in neuronal plasticity, postsynaptic development, mushroom spine density, memory, neuronal survival, caspase activation and dopaminergic neuron degeneration [66–69]. Selective knockdown of ATF4 impairs hippocampal LTP *in vitro* and *in vivo* [68] and as noted above, pharmacological inhibition of eIF2α dephosphorylation rescues cortico-striatal LTD defects in DYT1 KI mice [26]. Notably, eIF2α signaling was not among the top functional pathways in C54Y mice, and these mice exhibited reduced LTP but normal LTD. This highlights the presence of a synaptic plasticity defect in both dystonia models, but with genotype-driven differences as discussed earlier. Furthermore, these data suggest intriguing similarities between the different dystonia models that deserve further study, as there are other IPA and GO-enriched pathways in both genotypes that impinge on synaptic plasticity. These include LTD and LTP themselves in the ΔExon2 mice, the mTOR translation control pathway [70,71], Dopamine-DARPP-32 feedback in cAMP signaling [72], and G-protein/second messenger/cAMP gene groups.

Finally, we identify deficits in neuritogenesis *in vitro* as a possible convergence point between *Tor1a* and *Thap1* mutations. Mutant TorsinA inhibits neurite extension in cultured cells [73] and DYT1 mice have thinner and less complex dendrites in Purkinje cells and striatal medium spiny neurons [42,43]. The deficit in neuritogenesis is present in both ΔExon2 and C54Y striatal neurons. Many of the same highlighted GO and IPA pathways that could alter synaptic plasticity may also contribute to defective neuritogenesis, including the overlapping GO neurite projection terms and IPA Axonal Guidance Signaling, eIF2α/ATF4 pathway [74], Rho GTPase signaling, and G-protein and cAMP signaling pathways [75]. The fact that in 9-week-old mice, basal synaptic efficiency was enhanced in corticostriatal inputs of *Thap1*^*C54Y*^ mice yet normal in *Thap1*^*+/-*^ mice, suggests that any effects on neuritogenesis that persist into adulthood might be offset by additional synaptic changes. Future studies will need to address the temporal and biological relationship between the neurodevelopmental and plasticity phenotypes uncovered in this study.

### Additional insights into *THAP1*/DYT6 neurobiology

Other disordered pathways previously implicated in DYT1 and observed in the study reported herein are related to mitochondrial dysfunction [76,77] and lipid metabolism [78,79]. Interestingly, many of the *Thap1* DEGs which are included in the Emory University and Mount Sinai Genetic Testing Movement Disorders and Neuromuscular Disease Panels are also linked to those biological processes, suggesting that lipid metabolism and mitochondrial function may deserve further investigation in different forms of dystonia. Furthermore, torsins have recently been implicated as essential regulators of cellular lipid metabolism [78].

In conclusion, using an unbiased transcriptomic analysis in two brain regions from two mouse models of DYT6, we identified eIF2α dysregulation as a potential point of convergence between different forms of dystonia, possibly through its influence on key homeostatic neurodevelopmental events. The identification of similar eIF2α dysregulation and synaptic plasticity defects as previously described in DYT1 mice and rats in the DYT6 animals is a key convergence of biological mechanisms among inherited dystonias, perhaps adding the group of translational dysregulation-associated dystonias (DYT1, DYT3, DYT6, and perhaps DYT16 [24,26,80] to those linked to dopamine dysfunction (DYT5, DYT11, DYT25) [7]. Moreover, the disordered post-synaptic DARPP-32/G-protein/cAMP signaling system(s) potentially overlaps with other dystonias, particularly DYT25 caused by mutations in *GNAL* [8], suggesting that there are multiple pathways which may contribute to this phenotype. The consolidation of multiple types of dystonia into specific pathogenic mechanisms could facilitate focused research into the etiology of dystonia and the rational design of targeted therapies applicable to this group of movement disorders.

## Materials and methods

### Ethics statement

Experimental procedures were carried out in compliance with the United States Public Health Service's Policy on Humane Care and Use of Experimental Animals and were approved by the Institutional Animal Care and Use Committee (IACUC) at Icahn School of Medicine at Mount Sinai (Protocol #07–0483).

### Animals

The *Thap1*^*C54Y/+*^ and *Thap1*^*+/-*^ mice used in this study were congenic C57Bl. The generation of the original mice has been previously provided in detail in Ruiz *et al*. [18] and as the mutations are early embryonic lethal, breeding strategy was always heterozygote X WT. Animals were maintained on a 12:12 light: dark cycle with ad libitum access to food and water throughout the course of the entire experiment.

### Real-time qPCR, library preparation, and sequencing

Postnatal day 1 (P1) pups were euthanized by decapitation, the brain was dissected, snap-frozen striatal and cerebellar tissues were homogenized in QIAzol Lysis Reagent (Qiagen). Total RNA purification was performed with the miRNeasy mini kit (Qiagen), and was carried out according to the manufacturer’s instructions. Five hundred nanograms of RNA were reversed-transcribed using the High Capacity RNA-to-cDNA Kit (Applied Biosystems, Foster City, CA, USA). The cDNA solution was subjected to real-time qPCR in a Step-One Plus system (Applied Biosystems) using the PerfeCTa SYBR Green FastMix ROX (Quanta BioSciences). Quantitative PCR consisted of 40 cycles, 15 s at 95°C and 30 s at 60°C each, followed by dissociation curve analysis. Primer sequences can be found in S8 Table.

Total RNA from P1 striatal and cerebellar tissues (2–3 μg/ sample) were submitted for further processing to the Genomics Core Facility at the Icahn School of Medicine at Mount Sinai. Sequencing libraries were prepared with the TruSeq RNA Sample Prep Kit v2 protocol (Illumina, San Diego, CA, USA). Briefly, ribosomal RNA was removed using the Ribo-Zero rRNA Removal Kit (Human/Mouse/Rat) (Illumina), the remaining RNA fragmented and cDNA synthesized with random hexamers, end-repaired and ligated with appropriate adaptors for sequencing. After size selection and purification using AMPure XP beads (Beckman Coulter, CA, USA), 6 bp barcode bases were introduced at one end of the adaptors. The size and concentration of the RNA-Seq libraries was measured by Bioanalyzer and Qubit fluorometry (Life Technologies, Grand Island, NY, USA), and the rRNA-depleted libraries sequenced on the Illumina HiSeq 2500 System with 100 nucleotide paired-end reads.

### RNA-Seq data analysis

Fastq files were aligned to the Ensembl release 88 (GRCm38.75) version of Human Reference genome (mm10) [81], using STAR read aligner [82]. Accepted mapped reads were summarized separately to gene and exon levels using the featureCounts function of subread [83,84], and used to generate gene and exon count matrices. We examined gene expression data for each sample, and found that the total number of mapped reads [Total Reads (Mean): 39,708,979.5; Uniquely Mapped Reads (Mean): 36,244,152.41] were similar across all samples (S10 Table). There were no obvious outlier samples on visual inspection of principal component analysis or hierarchically clustering of gene expression, and all samples were retained for downstream analyses.

### Differential gene expression analysis

For each of the primary comparisons within the study, the assembled count matrix was filtered to remove transcripts without any counts in any sample. Counts were adjusted by total library size and normalized using DESeq2 [85]. P-values were adjusted using the Benjamini-Hochberg method [86]. Due to the small number of DEGs present in striatum and cerebellum of *Thap1*^*C54Y/+*^ mice, p-value of 0.05 was used as cut off.

### Mitochondrial respiratory complex I activity

Mitochondrial respiratory complex I activity assay (ab109721, Abcam) was performed, utilizing striatal and cerebellar protein lysates from P1 *Thap1*^*+/-(ΔExon2)*^ and WT mice (n = 4 per genotype), according to manufacturer's instructions.

### Treatment with tunicamycin

Two subcutaneous injections of 3 μg/g tunicamycin (T7765, Sigma-Aldrich) diluted in 150mM dextrose (or dextrose-only control) were applied two hours apart on postnatal day 4 as described [30]. Mice were euthanized 24 hours later, the striatum and cerebellum dissected and snap frozen.

### Western blotting

Snap-frozen striatal and cerebellar tissues were homogenized in RIPA buffer with N-ethylmaleimide and protease/phosphatase inhibitors as described previously [24,87]. Protein concentrations were determined using the BCA assay (23225, ThermoFisher Scientific), 30 μg protein lysates were resolved in 10% or 12% Bis/Trisacrylamide gels (BioRad), transferred to nitrocellulose membranes and western blot completed and quantified using the following antibodies: ATF4 / CREB-2 (1:200; sc-200, Santa Cruz), BiP (1:1000; 3177, Cell Signaling), PERK (1:1000; 5683, Cell Signaling), p-PERK (1:1000; 3179, Cell Signaling), eIF2α (1:200; sc-11386, Santa Cruz), p-eIF2α (1:1000; 9721, Cell Signaling), CHOP (1:500; sc-7351, Santa Cruz), mTOR (1:1000; 9862, Cell Signaling), p-eIF4B (Ser406) (1:1000; 5399, Cell Signaling) and GAPDH (1:1000; sc-32233, Santa Cruz). All primary antibodies were incubated in TBS-Tween 5% milk for overnight at 4°C. Membranes were then washed in TBS-Tween. Secondary antibodies anti-rabbit IgG–HRP (PI-1000, Vector Laboratories) or anti-mouse IgG-HRP (PI-2000, Vector Laboratories) were used (1: 5,000) in TBS-Tween 5% milk for 1 hour at room temperature. Immunoreactive proteins were visualized using either Pierce ECL Western Blotting Substrate (32106, ThermoScientific) or Amersham ECL Prime Western Blotting Detection Reagent (RPN2232, GE Health) on a Fujifilm LAS4000 imaging device.

### Electrophysiology

Nine-week-old mice were anesthetized with isoflurane, their brains rapidly removed from the skull and placed in ice-cold modified solution (aCSF) containing (in mM): 215 sucrose, 2.5 KCl, 1.6 NaH_2_PO_4_, 4 MgSO4, 1 CaCl_2_, 4 MgCl_2_, 20 glucose, 26 NaHCO_3_ (pH = 7.4, equilibrated with 95% O_2_ and 5% CO_2_) and 250 μm thick coronal slices containing the striatum prepared with a Vibratome VT1000S (Leica Microsystems), incubated at 31°C for 30 min and then at room temperature for ≥ 1h in normal aCSF containing (in mM): 120 NaCl, 3.3 KCl, 1.2 Na_2_HPO_4_, 26 NaHCO_3_, 1.3 MgSO_4_, 1.8 CaCl_2_, 11 glucose (pH = 7.4 equilibrated with 95% O_2_ and 5% CO_2_). Hemi-slices were transferred to a recording chamber constantly oxygenated and perfused with aCSF at ~4mL/min using a peristaltic pump (Masterflex C/L); experiments were performed at 28.0 ± 0.1°C. Recordings were acquired with a GeneClamp 500B amplifier (Axon Instruments) and Digidata 1440A (Molecular Devices). All signals were low-pass filtered at 2 kHz and digitized at 10 kHz. For field EPSP recordings, a patch pipette was fabricated on a micropipette puller (Sutter Instruments), filled with normal aCSF, and placed in the dorsomedial striatum for LTP or dorsolateral striatum for LTD. A concentric bipolar electrode (FHC) was positioned immediately above the corpus callosum. Before and after HFS, the stimulus intensity was set evoke an EPSP that was 50% of the maximal obtainable response. During HFS, stimulus intensity was increased to evoke a maximal response. LTD or LTP was induced by the following high-frequency stimulation (HFS) protocol: four 1-s duration, 100 Hz trains, separated by 10 s. mGluR LTD was induced by a 5-min bath application of 100 μM DHPG [(S)-3,5-Dihydroxyphenylglycine]. Sal003, when present, was applied for 5–10 min before DHPG, and washed out with DHPG. Gabazine, when present, was applied at 10 μM for at least 20 min before the delivery of HFS, and remained in the superfusate for the remainder of the recording. Square-wave current pulses (100 μs pulse width) were delivered through a stimulus isolator (Isoflex, AMPI). Paired-pulse ratio was measured by delivering two stimuli at 20, 50, 100 ms inter-stimulus intervals. Each inter-stimulus interval was repeated three times and the resulting EPSPs were averaged.

### Quantification of neurite outgrowth

Primary striatal cultures were prepared as described [10], fixed with 4% paraformaldehyde 48 hours after plating, and processed for immunostaining following our published protocol [14] using the following primary antibodies: Tau (1:500, MN1000, ThermoFisher Scientific), TUJ1 (1:250, sc-51670, Santa Cruz), and MAP2 (1:1,000; AB5622, Millipore). Cells were visualized under an Olympus IX51 inverted fluorescent microscope.

NeuriteTracer, a neurite tracing plugin for the freely available image-processing program ImageJ was used to analyze fluorescence microscopy images of neurites and nuclei of cultured primary neurons. The plugin was used to count neuronal nuclei, and traces and measure neurite length as described [88]. Ten randomly selected images of each neuronal culture type were processed. DAPI staining was employed as a nuclear stain. The average length was obtained by dividing the total length of the traces by the number of nuclear counts.

### Statistical analyses

GraphPad software (GraphPad Prism 5) was used to perform Student's t-tests for the qPCR and western blot densitometry, two-way ANOVAs followed by Tukey’s *post hoc* tests were used to analyze the tunicamycin western blot densitometry. Statistical differences in neurite length / outgrowth were assessed by ANOVA with Student’s *post hoc* t-test. Statistical significance was deemed to be achieved if P < 0.05. Values are presented as mean ± SEM. Electrophysiological data were analyzed by one-way ANOVAs followed, where appropriate, by Newman-Keuls *post hoc* tests.

### Accession numbers

All next generation sequencing data are deposited in NCBI-Gene Expression Omnibus database and are accessible through GEO Series accession number GSE98839. The accession number for human ENCODE ChIP-Seq data from K562 cells used in the manuscript for THAP1 is GEO: GSM803408. The accession number for the mouse embryonic stem (ES) cells ChIP-Seq data for Thap1 used in the manuscript is GEO: GSE86911.

## Supporting information

S1 Fig Basal expression of key constituents from the eIF2α, and mTOR signaling pathways in the striatum and cerebellum of *Thap1*^*+/-*^ vs WT pups at postnatal day 1. Full western blots of striatal and cerebellar lysates obtained from *Thap1*^*+/-*^ and WT littermates for expression of the indicated proteins. * denotes nonspecific band. CHOP bands are compared to the lower GAPDH panel whereas all others are compared to the highest GAPDH panel.
(TIF)

S2 Fig Effects of *Thap1* mutations on corticostriatal LTD are not mediated by GABA signaling. The GABA-A antagonist gabazine (10μM) was present throughout the recordings. Left: Time-course; representative traces show baseline (dashed lines) and 40 min post HFS (solid lines), with colors corresponding to the time-course graph. Calibration: 1 mV / 5 ms. Right: Summary data over final 5 min of recording (mean ± sem). LTD, measured over the final 5 min, was reduced in slices from *Thap*^*+/-*^ mice, and enhanced in slices from *Thap1*^*C54Y/+*^ mice (ANOVA followed by Neman-Keuls *post hoc* tests). See also S9 Table.
(TIF)

S3 Fig Western blot analysis of **(A)** striatal and **(B)** cerebellar lysates from *Thap1*^*+/-*^ and WT littermates for p-eIF2α/eIF2α, and p-eIF2α/GAPDH were performed 24 hrs after subcutaneous tunicamycin (TM) diluted in 150mM dextrose (or dextrose-only control; DEX). Data are presented as means ± SEM; n = 3 for each genotype and region; data normalized to WT (dextrose-only) controls. Statistical differences were assessed by two-way ANOVAs with Tukey’s *post hoc* tests. See also S9 Table.
(TIF)

S1 Table Differential gene expression analysis in the striatum of *Thap1*^*+/-*^ vs WT mice using IPA platform analysis: Canonical Pathways, Diseases and Biofunctions, DEGs (LOG2 Ratios, p-values), and DAVID Gene Ontology (GO) terms (biological process). (XLSX)

S2 Table Differential gene expression analysis in the striatum of *Thap1*^*C54Y/+*^ vs WT mice using IPA platform analysis: Canonical Pathways, Diseases and Biofunctions, DEGs (LOG2 Ratios, p-values), and DAVID Gene Ontology (GO) terms (biological process). (XLSX)

S3 Table Differential gene expression analysis in the cerebellum of *Thap1*^*+/-*^ vs WT mice using IPA platform analysis: Canonical Pathways, Diseases and Biofunctions, DEGs (LOG2 Ratios, p-values), and DAVID Gene Ontology (GO) terms (biological process). (XLSX)

S4 Table Differential gene expression analysis in the cerebellum of *Thap1*^*C54Y/+*^ vs WT mice using IPA platform analysis: Canonical Pathways, Diseases and Biofunctions, DEGs (LOG2 Ratios, p-values), and DAVID Gene Ontology (GO) terms (biological process). (XLSX)

S5 Table Putative THABs motifs in DEGs overlapping in the striatum and cerebellum of *Thap1*^*+/-*^ and *Thap1*^*C54Y/+*^ vs WT mice. In silico analysis was used to identification putative THABS motifs in 1KB of sequence upstream of the translation start codon. Putative THABs motif indicated by “+” symbol.
(XLSX)

S6 Table Cross-match of the lists of DEGs found to be dysregulated in either of the Thap1 mutant striatum or cerebellum (*Thap1*^*C54Y/+*^ or *Thap1*^*+/-*^ vs WT) with disease genes that have been previously linked to dystonia or other movement disorders, using the Emory University dystonia sequencing panel and the Mount Sinai Genetic Testing Laboratory Movement Disorders and Neuromuscular Disease Panel. (XLSX)

S7 Table List of brain specific cell types and their respective DEGs in the striatum and cerebellum of *Thap1*^*+/-*^ or *Thap1*^*C54Y/+*^relative to WT mice. (XLSX)

S8 Table List of primer sequences for RT-qPCR. Primer sequences for the constituents of eIF2α signaling pathway, and the top 5 DEGs from the striatum and cerebellum of *Thap1*^*+/-*^ or *Thap1*^*C54Y/+*^ relative to WT.
(XLSX)

S9 Table Table detailing the statistical analyses for all significant data, including tests, and *post hoc* analysis where appropriate, for Figs 3–7, and S2 Fig. (XLSX)

S10 Table Summary table of mapped reads for each RNA-Seq sample used in the analysis including total reads, uniquely mapped reads, and fraction mapped reads. (XLSX)

S11 Table **Cross-match lists of DEGs found to be dysregulated in either of the Thap1 mutant striatum or cerebellum (*Thap1*^*C54Y/+*^ or *Thap1*^*+/-*^ vs WT) with THAP1 publicly available ChIP-Seq datasets from (A) Thap1 mouse embryonic stem cells, and (B) human ENCODE K562 cells.** The highest ranking biological functions as identified by DAVID GO for shared genes between the *Thap1*^*+/-*^ cerebellum and the (C) Thap1 mouse embryonic stem cells, and (D) human ENCODE K562 cells are shown.
(XLSX)
