# Supplementary figures and images for: Mutations in THAP1/DYT6 reveal that diverse dystonia genes disrupt similar neuronal pathways and functions
Source: PLoS Genet. 2018 Jan 24;14(1):e1007169. doi: 10.1371/journal.pgen.1007169 (PMC5798844; doi:10.1371/journal.pgen.1007169)

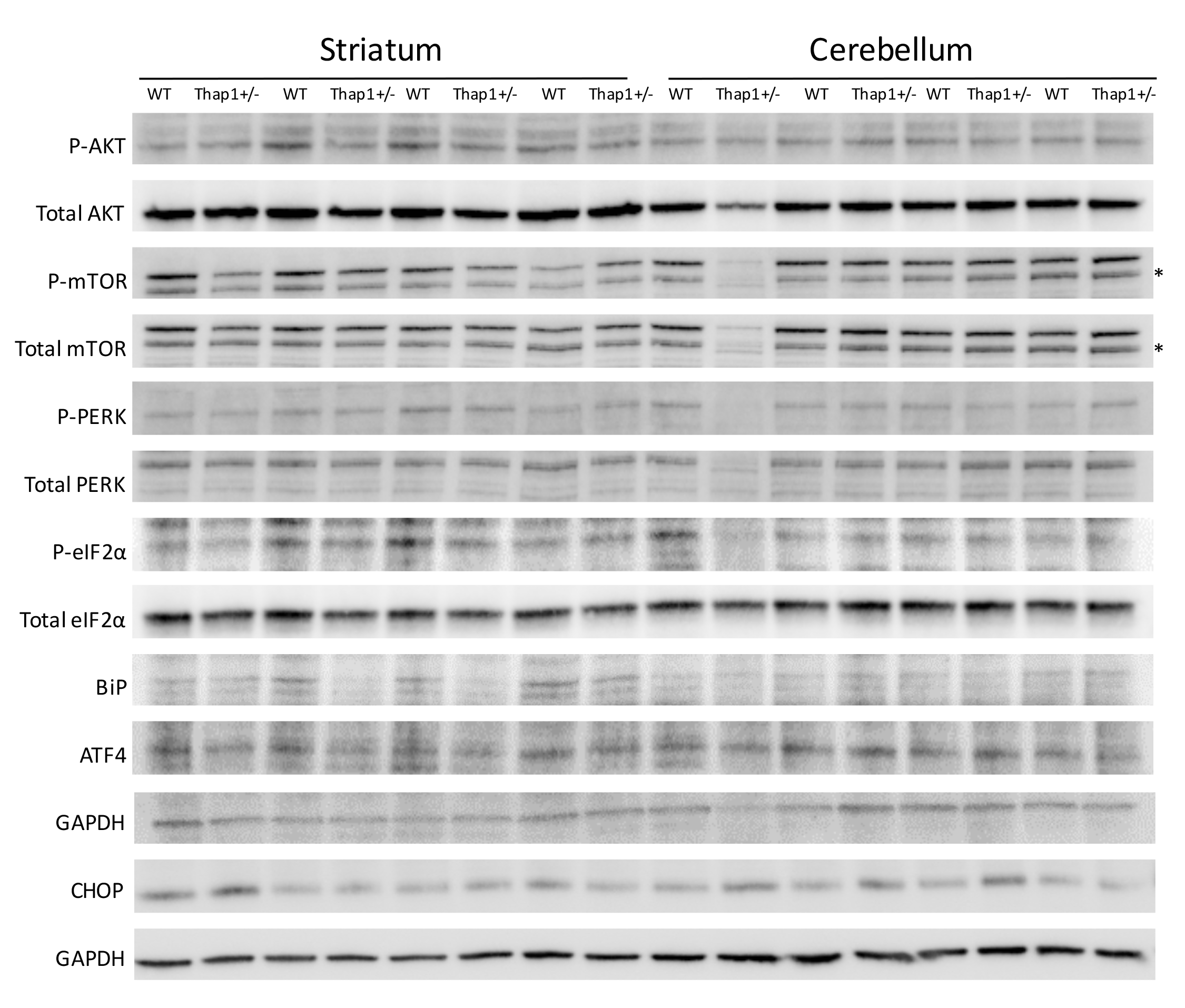

Supplement: S1 Fig — Full western blots of striatal and cerebellar lysates obtained from Thap1+/- and WT littermates for expression of the indicated proteins. * denotes nonspecific band. CHOP bands are compared to the lower GAPDH panel whereas all others are compared to the highest GAPDH panel. (TIF) [file pgen.1007169.s001.tif]

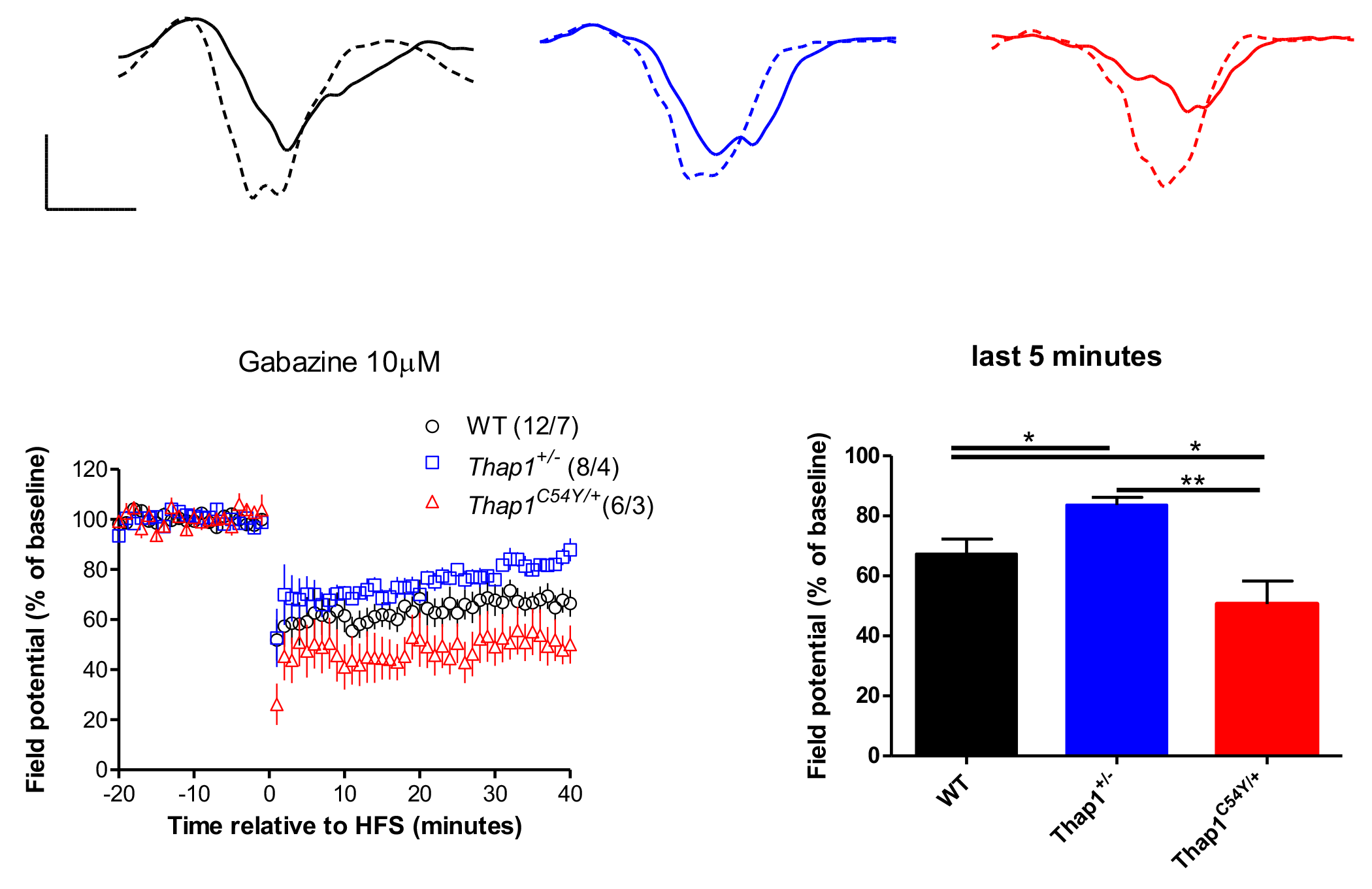

Supplement: S2 Fig — The GABA-A antagonist gabazine (10μM) was present throughout the recordings. Left: Time-course; representative traces show baseline (dashed lines) and 40 min post HFS (solid lines), with colors corresponding to the time-course graph. Calibration: 1 mV / 5 ms. Right: Summary data over final 5 min of recording (mean ± sem). LTD, measured over the final 5 min, was reduced in slices from Thap+/- mice, and enhanced in slices from Thap1C54Y/+ mice (ANOVA followed by Neman-Keuls post hoc tests). See also S9 Table. (TIF) [file pgen.1007169.s002.tif]

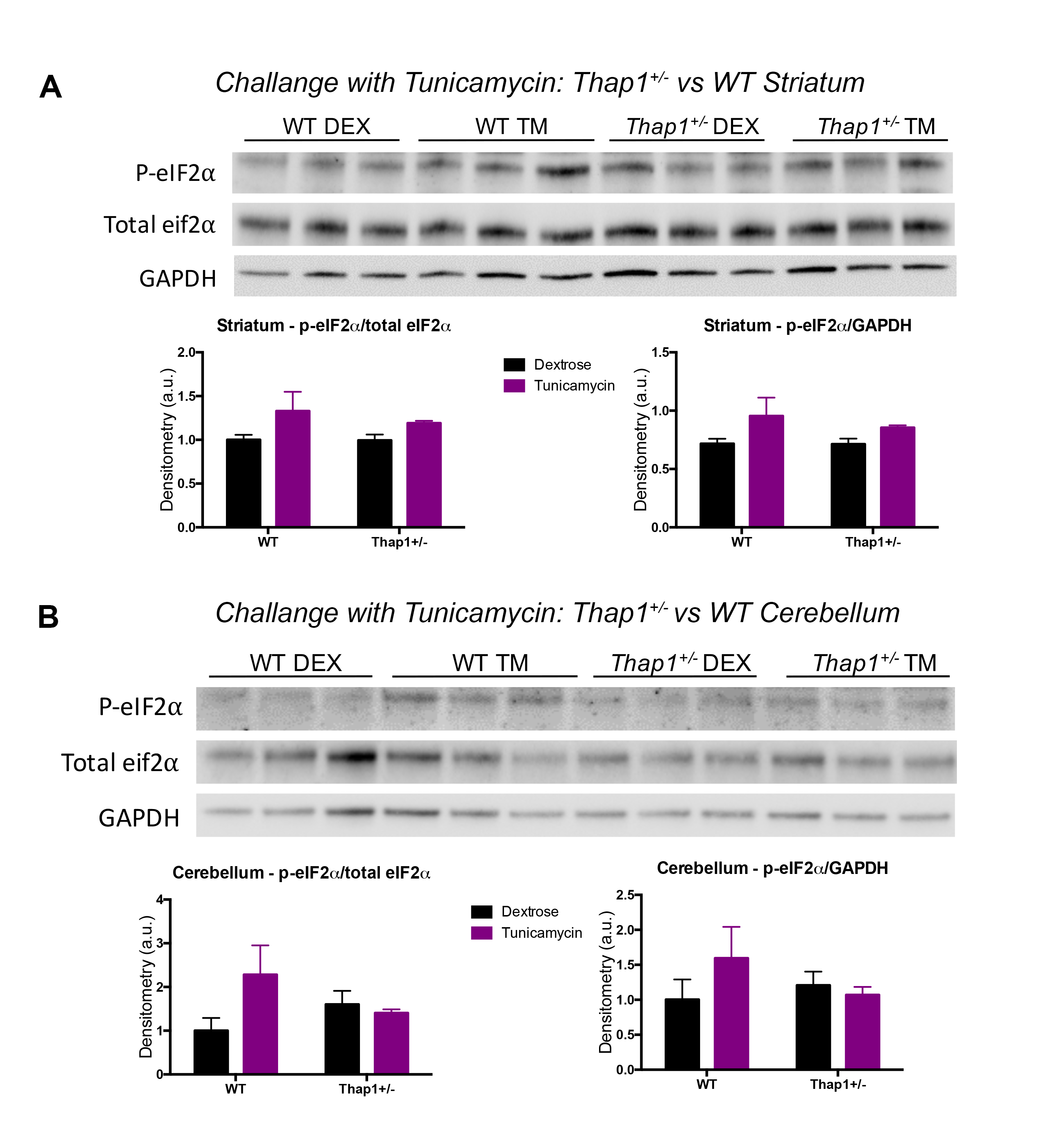

Supplement: S3 Fig — Western blot analysis of (A) striatal and (B) cerebellar lysates from Thap1+/- and WT littermates for p-eIF2α/eIF2α, and p-eIF2α/GAPDH were performed 24 hrs after subcutaneous tunicamycin (TM) diluted in 150mM dextrose (or dextrose-only control; DEX). Data are presented as means ± SEM; n = 3 for each genotype and region; data normalized to WT (dextrose-only) controls. Statistical differences were assessed by two-way ANOVAs with Tukey’s post hoc tests. See also S9 Table. (TIF) [file pgen.1007169.s003.tif]
